# Juvenile Dermatomyositis: Association between Nail Fold Capillary End Row Loops Area Under the Curve and Disease Damage Indicators

**DOI:** 10.21203/rs.3.rs-3235841/v1

**Published:** 2023-08-21

**Authors:** Amer Khojah, Gabrielle Morgan, Marisa S. Klein-Gitelman, Lauren M. Pachman

**Affiliations:** Umm Al-Qura University College of Medicine; Ann and Robert H Lurie Children’s Hospital of Chicago; Ann and Robert H Lurie Children’s Hospital of Chicago; Ann and Robert H Lurie Children’s Hospital of Chicago

**Keywords:** Juvenile Dermatomyositis, Nailfold vasculature, Disease Activity Scores, area under the curve, lipodystrophy

## Abstract

**Background:**

Juvenile Dermatomyositis (JDM) is a rare autoimmune disease characterized by skin and muscle inflammation. The loss of nail fold capillary end row loops (ERL) is evidence of small vessel involvement in JDM. This study aimed to examine the association of ERL over the disease course and evidence of disease damage.

**Methods:**

We analyzed data from 68 initially treatment-naïve JDM children who had been observed for at least five years with multiple ERL density assessments. The JDM disease courses were categorized into monocyclic short, monocyclic long, polycyclic, and chronic. The ERL capillary count was cumulatively evaluated using the area under the curve (AUC) method.

**Results:**

The mean ERL density for the treatment-naive JDM was significantly lower than that of their healthy controls (4.8±1.6 /mm vs. 7.9±0.9 /mm; p <0.0001). The ERL AUC was significantly lower in children with chronic disease course compared to those with monocyclic short (p =0.001) or monocyclic long disease course (p =0.013). JDM patients with lipodystrophy had lower ERL AUC than those without lipodystrophy (p =0.04). There was no association between ERL AUC and calcifications or fractures.

**Conclusion:**

Persistently decreased ERL capillary density, evident by low ERL AUC, is associated with chronic disease course and lipodystrophy in JDM. Despite medical therapy, the mean ERL count remained below normal even after five years, particularly in polycyclic and chronic cases. Therefore, the goal of restoring normal capillary density in children with JDM might be challenging and require novel therapeutic strategies targeting their underlying endothelial dysfunction.

## Introduction

Juvenile Dermatomyositis (JDM) is a systemic pediatric autoimmune disease characterized by skin and muscle inflammation (1). Although it is the most common pediatric inflammatory myopathy, with an incidence rate of approximately 3.2 cases per million children in the United States, JDM remains a rare disease (2). Its etiology is not entirely understood, but it is believed to result from a combination of genetic predisposition and environmental factors, such as viral infections and exposure to ultraviolet rays (3, 4).

One of the key features of JDM is the loss of nailfold capillary end row loops (ERL), which can be evaluated at the bedside using capillaroscopy (5–7). The loss of capillary ERL indicates small vessel vascular injury, which is further supported by elevated levels of von Willebrand factor antigen (vWF:Ag) in the treatment-naïve JDM patients (8). The reduction in ERL is linked to lower bioavailability of oral prednisone compared to IV methylprednisolone (KRS), which reduces the effectiveness of oral treatment (9). Inadequate treatment of JDM can lead to a higher risk of complications such as calcinosis, deposition of insoluble calcium salts in the skin and subcutaneous tissue, and lipodystrophy (10, 11). Therefore, understanding the association between ERL density and disease progression is important.

This study aims to examine the association between ERL density over time (5 years) using the area under the curve (AUC) method and various diseases courses (monocyclic short, monocyclic long, polycyclic, and chronic) as well as indicators of disease damage (lipodystrophy, calcification, and fractures).

## Methods

### Subjects

This retrospective chart review study (IRB# 2012–14858) was conducted at Ann & Robert H. Lurie Children’s Hospital of Chicago. We included all subjects with JDM diagnosis based on Bohan and Peter criteria who had at least five years of follow-up data and had at least four ERL assessments at a prespecified time point (0,6,12,24,36,48, and 60 months). Patients with overlap syndrome were excluded from the analysis. The JDM disease activity was evaluated using standardized scoring systems– the Disease Activity Score (DAS) (12) and Childhood Myositis Assessment Scale (CMAS) (13).

#### Disease course:

Children with JDM were categorized into four distinct disease courses according to their treatment response: A) monocyclic short: if the child completed therapy within the first 36 months without a subsequent disease flare; B) monocyclic long: if the child completed therapy after 36 months without a subsequent disease flare; C) polycyclic: if the child had completed treatment but had a subsequent relapse of disease requiring re-initiation of medication; D) chronic: no clinical resolution within 60 months.

#### Nailfold capillary ERL studies:

Standardized images of the nailfold area were obtained using a Nikon Coolpix p6000 digital camera equipped with a Dermlite2 ProHR. Each patient’s mean ERL/mm was calculated by averaging the ERL/mm of the eight fingers (5).

#### ERL area under the curve calculation

**Figure 1:**
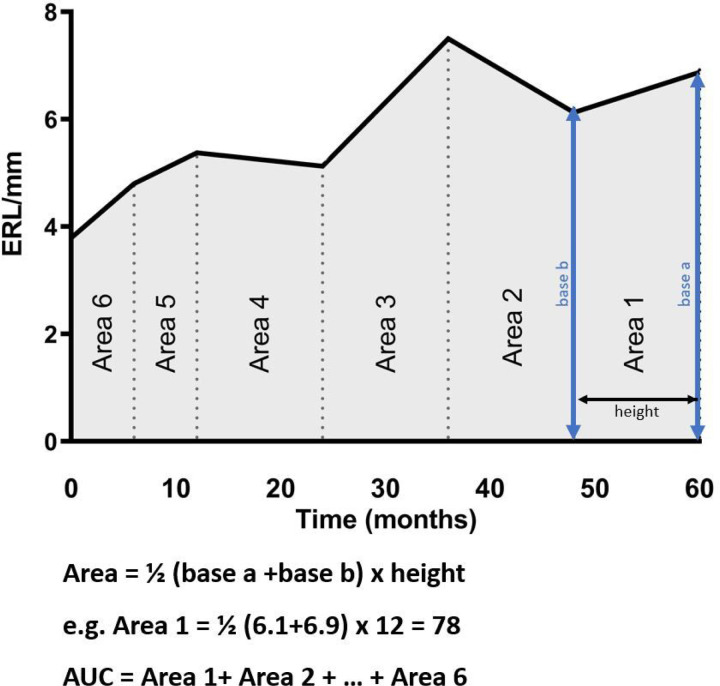
Area under the curve (AUC) calculation by GraphPad Prism. First, the curve was created by plotting the ERL data over time. Then, Prism divides AUC into multiple small trapezoid areas, which are measured individually, using the trapezoid rule [area = ½ (base a + base b) x height], and added up to get the total AUC.

GraphPad Prism was used to calculate the AUC to measure the ERL cumulatively across the study duration. First, the curve was created by plotting the ERL data over time. Then, Prism divides AUC into multiple small trapezoid areas, which are measured individually, using the trapezoid rule [area = ½ (base a + base b) x height], and added up to get the total AUC (Fig. 1).

### Statistical analysis

The Person’s correlation coefficient was utilized to assess the relationship between ERL at diagnosis and ERL AUC. The student t-test was used to compare the mean ERL AUC of subjects with and without signs of disease damage. Statistical analyses were conducted using IBM SPSS Statistics^®^ and GraphPad Prism^®^ version 9.4.1 was utilized to generate the figures.

## Results

The study included 68 treatment-naive JDM children, the majority of whom were female (84%). The racial and ethnic distribution was as follows: 75% Caucasian, 19% Hispanic, 1.5% Asian, and 3% African American. Their MSAs (Myositis-specific antibodies) were as follows: 41% P155/140+, 3% MJ+, 7.5% Mi-2+, 3% MDA-5+, 7.5% multiple MSAs, and 31% MSA negative. The mean age of onset for JDM was 6 ± 3.1 years, and the mean duration of untreated disease was 9.6 ± 10.2 months. The initial disease activity scores were 11.0 ± 3.6 for DAS total, 5.9 ± 1.5 for DAS skin, and 5.1 ± 2.9 for DAS muscle, and CMAS score was 37 ± 10.3. The disease course distribution was as follows: 17.6% monocyclic short, 42.6% monocyclic long, 20.6% polycyclic, and 19.1% chronic.

**Figure 2:**
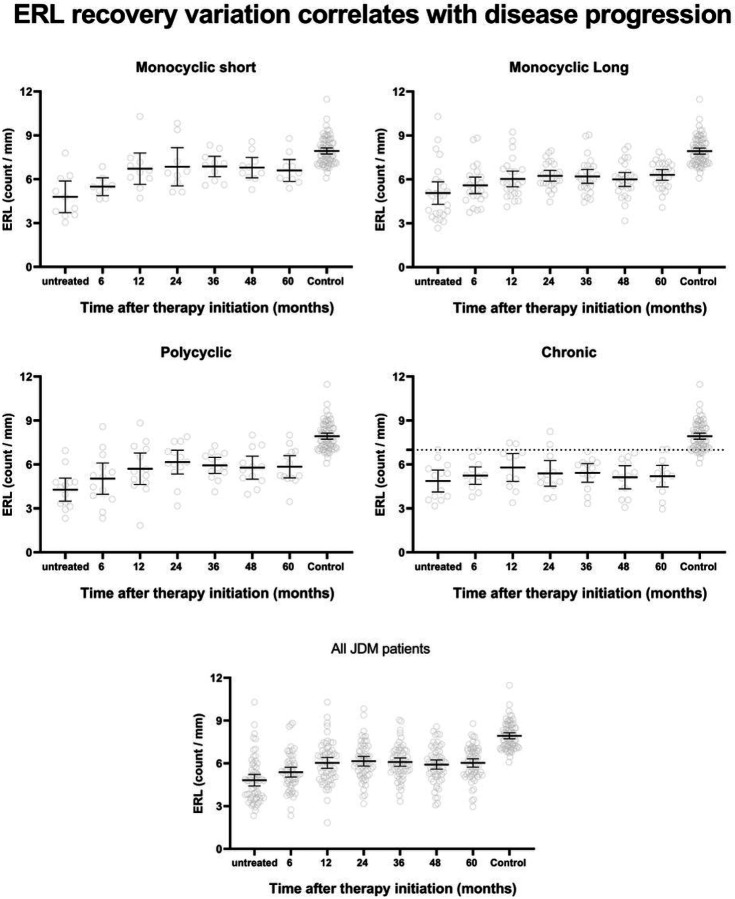
Changes in ERL capillary count over time (5 years) by disease course categories. The rate of improvement varied depending on the different disease courses with monocyclic short showing faster recover than the other groups.

The mean ERL count for treatment-naive JDM was 4.8 ± 1.6/mm, which is significantly lower than the healthy control 7.9 ± 0.9/mm (p < 0.0001). Despite the improvement in mean ERL count over time, it remained below the expected normal level (6.1–9.7/mm), even after five years of medical therapy (Fig. 2). The rate of improvement varied depending on the different disease courses, with monocyclic short showing more change than the other groups (4.8 ± 1.5/mm at baseline vs. 6.7 ± 1.5/mm at 12 months, p = 0.038 paired T-test).

**Figure 3:**
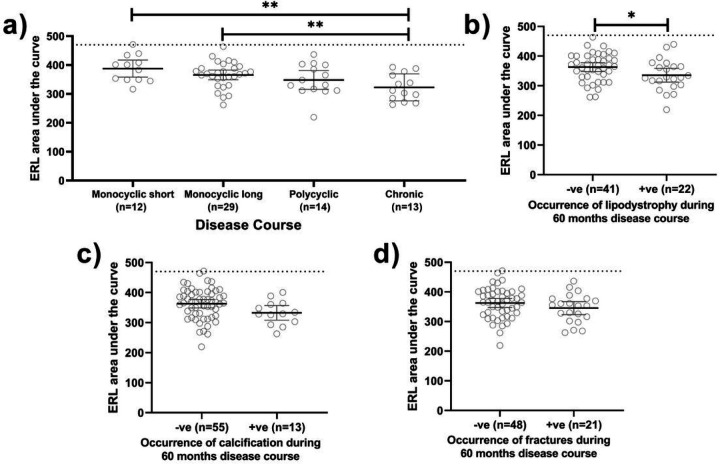
Disease courses and complications vs. ERL area under the curve (AUC). A) There is a significant difference between the AUC for monocyclic short vs chronic course as well as a significant difference between monocyclic long vs chronic disease course, both p<0.01. B) Lipodystrophy of any type (generalized, partial, or localized) has lower ERL AUC, p=0.04 than JDM without lipodystrophy. C) There is no association of ERL AUC with calcifications. D) There is no association of ERL AUC with fractures.

To evaluate the accumulative effects of chronic ERL capillary loss, the ERL area AUC was calculated for each patient (Fig. 1). Although there was a positive correlation between the initial ERL count and ERL AUC, the correlation was not strong (r^2^ = 0.18, p = 0.001). There was a significant difference between the ERL AUC for monocyclic short vs. chronic (389 ± 46.46 vs. 313 ± 46.69, p = 0.001) and monocyclic long vs. chronic (359 ± 44.53 vs. 313 ± 46.69, p = 0.013) (Fig. 3a). Finally, the relationship between ERL AUC and indicators of disease damage (lipodystrophy, calcification, and fractures) was evaluated. JDM patients with lipodystrophy had a lower ERL AUC than those without lipodystrophy (335.7 ± 52.52 vs ± 47.13, p = 0.04) (Fig. 3b). However, ERL AUC had no significant association with calcifications (Fig. 3c) or fractures (Fig. 3d).

## Discussion

This study provides insights into the association between endothelial dysfunction evident by decreased ERL count and disease progression in JDM. Consistent with previous studies, we observed a significantly lower mean ERL density in untreated JDM children compared to healthy controls (6, 7). This finding supports the concept of endothelial involvement in JDM pathophysiology (3). Furthermore, the reduction in ERL count often correlates with the severity of skin disease and/or muscle weakness (6, 14), suggesting its possible role as a potential biomarker for disease activity. Few studies have evaluated the changes in capillary density and disease activity longitudinally over the disease course (14, 15). We observed variations in the rate of improvement in ERL count among different disease courses. Monocyclic disease courses show improvement of the ERL capillary count at a faster rate than chronic disease. This finding suggests that the rate of improvement in ERL count may be a more critical factor than the initial ERL count in predicting disease course and outcome.

Despite medical therapy, the mean ERL count in JDM patients remained below normal even after five years of treatment, particularly in the polyphasic and chronic disease courses. This implies that the restoration of capillary density might be challenging to achieve and may require novel therapeutic strategies to target endothelial dysfunction effectively (16).

Additionally, we utilized the AUC method to evaluate the cumulative change in ERL capillary density over the study period. Our results demonstrated a correlation between ERL AUC and a more chronic disease course, as well as the presence of complications such as lipodystrophy. Of note, we found a positive correlation between the initial ERL count and ERL AUC, albeit not particularly strong. This suggests that factors other than the initial capillary density, including MSAs, may contribute to the cumulative capillary loss. For example, it has been shown JDM with anti-P155/140 tend to have lower ERL capillary count and are less likely to have a monophasic disease course (5). However, our study was not powered enough to investigate the impact of different MSAs on ERL AUC.

Circulating endothelial cells and markers of endothelial injury, such as vWF:Ag, and thrombomodulin, aree elevated in JDM, providing further evidence of endothelial involvement in the disease pathophysiology (8, 16, 17). B cell activation and expansion as well as the formation of anti-endothelial cell antibodies have been demonstrated in JDM (18–21), suggesting another potential mechanism for endothelial cell injury. Soluble adhesion molecule markers, such as ICAM-1, ICAM-3, and VCAM1, and inflammatory cytokine and markers like neopterin have been used as possible biomarkers of vasculopathy in JDM (22–24). These findings highlight the complex interplay between immune dysregulation and endothelial dysfunction in JDM, requiring further investigation to elucidate the underlying mechanisms. Furthermore, it is important to recognize the clinical implications of microvascular injury in JDM as it can affect the gastrointestinal system (25). The reduced nailfold capillary density observed in JDM has been associated with impaired absorption of oral prednisone, potentially leading to suboptimal drug levels and ineffective treatment (9). Therefore, administering medications by the intravenous or subcutaneous routes might be preferred in patients with persistently low ERL counts.

In summary, persistently decreased ERL capillary density documented by low ERL AUC is associated with both a chronic disease course and lipodystrophy in JDM. Despite medical therapy, the mean ERL count remained below normal, even after five years, particularly in polycyclic and chronic cases. Therefore, restoring normal capillary density might be challenging and require novel therapeutic strategies targeting endothelial dysfunction.

## Abbreviations

JDM: Juvenile Dermatomyositis
ERL: end row loops
AUC: area under the curve
vWF:Ag: von Willebrand factor antigen
IV: intravenous
DAS: Disease Activity Score
CMAS: Childhood Myositis Assessment Scale
MSA: Myositis-specific antibodies
